# Association between characteristics of pain and stiffness and the functional status of patients with incident polymyalgia rheumatica from primary care

**DOI:** 10.1007/s10067-017-3730-6

**Published:** 2017-06-20

**Authors:** A. Cawley, J. A. Prior, S. Muller, T. Helliwell, S. L. Hider, B. Dasgupta, K. Barraclough, C. D. Mallen

**Affiliations:** 1 Research Institute for Primary Care and Health Sciences, Keele University, Newcastle, ST5 5BG UK; 2 Haywood Academic Rheumatology Centre, Haywood Hospital, Stoke-on-Trent, Staffordshire UK; 3 Southend University Hospital, Westcliff-on-Sea, UK; 4 Painswick Surgery, Painswick, Gloucestershire UK

**Keywords:** Cohort, Function, mHAQ, Modified health assessment questionnaire, Polymyalgia Rheumatica, Primary care

## Abstract

This paper aims to examine the relationship between different characteristics of pain and stiffness and the functional status of patients with newly diagnosed polymyalgia rheumatica (PMR). Baseline analysis of an inception cohort study was conducted. Patients aged ≥18 years, with a new diagnosis of PMR were recruited from 382 English general practices. Participants were mailed a baseline questionnaire, including separate pain and stiffness manikins and numerical rating scales (NRS), a question on their ability to raise their arms above their head and the modified Health Assessment Questionnaire (mHAQ) to examine participants’ functional status. Linear regression analysis, reported as regression co-efficients (95% confidence intervals (95% CI)), was used to assess the association of pain and stiffness with function, initially unadjusted and then adjusted for age, gender, deprivation status, smoking status, BMI, anxiety and depression. Six hundred fifty two patients responded to the baseline survey (88.5%). The majority (88.2%) reported no, or mild impairment in their functional status. Adjusted linear regression analysis demonstrated that high (NRS ≥8) pain (0.20 (95% CI 0.10–0.28)) or stiffness (0.18 (0.09–0.26)) ratings, an increasing number of sites of pain (0.18 (0.06–0.29)) or stiffness (0.19 (0.08–0.31)) and shoulder pain (0.18 (0.05–0.31)), stiffness (0.10 (0.01–0.20)) and difficulty raising arms above one’s head (0.19 (0.10–0.28)) were all associated with increased functional impairment. The majority of newly diagnosed PMR patients reported no or minimal functional difficulty. However, those who experience severe or widespread pain or stiffness often have significant functional limitation in performing their daily activities and may be a subset worthy of additional focus in primary care.

## Introduction

Polymyalgia rheumatica (PMR) is an inflammatory condition seen in patients aged over 50 years, with incidence increasing with age and peaking between 70 and 80 years. PMR is characterised by bilateral pain and stiffness in the shoulders, upper arms or pelvic region, with raised inflammatory markers and a rapid response to glucocorticoid treatment (commonly prednisolone in the UK). It is also often associated with constitutional symptoms such as fatigue, malaise and weight loss [1, 2].

Pain and stiffness are common in patients with PMR, and these symptoms are frequently used to denote the ‘classical’ presentation description of bilateral involvement of the shoulders and hips. Pain and stiffness can also be an indirect marker of functional ability, an outcome measure reported as more important to some patients than either specific pain or stiffness symptoms [3]. Functional status is an important indicator of patient health, both in isolation and with other outcome measures. This is positively correlated to C reactive protein (CRP) [4] and is a strong predictor of mortality in both the general population and for patients with rheumatoid arthritis (RA) [5]. Limitations in the patients’ ability to conduct activities of daily living can have significant negative impacts on self-management, drug adherence and mental health [6], presenting it as a potential marker of future adverse health outcomes.

Though pain, stiffness and functional limitations are not uncommon in patients with PMR, there is limited research into the relationship between these during the early stages of PMR. In a sample of patients with PMR from secondary care, Hutchings et al. found there to be a major reduction in the functional status of this group, as measured by the Health Assessment Questionnaire (HAQ) [6]. They showed that functional status was significantly below the expected norm for the general population and that pain and stiffness were most strongly associated with changes in functional status. However, patients referred to secondary care are likely to represent the more severe or atypical end of the disease spectrum compared to patients in primary care [7], where the majority of patients with PMR are managed after ruling out a differential diagnosis that would require further intervention of rheumatology [8].

Our aim was to examine the relationship between different characteristics of pain and stiffness and the functional status of newly diagnosed patients with PMR, hypothesising that increased pain or stiffness would be associated with a reduced ability to perform daily activities.

## Methods

### Study design and population

We examined patient reported baseline data from an inception cohort study of patients with newly diagnosed PMR in general practice. The methods of the PMR cohort study have been described elsewhere [9], but in summary, newly diagnosed PMR patients were recruited from 382 general practices from across England between June 2012 and June 2014. Patients agreeing to be contacted were mailed a postal questionnaire and consent form, with a reminder pack sent after 3 weeks for non-responders. Study approval was obtained from the Staffordshire Local Research Ethics Committee (Ref no: 12/WM/0021).

### Outcome measures

Participants were asked to rate the severity of their pain and stiffness using two separate 0–10 numerical rating scales (NRS), with 0 indicating no pain/stiffness and 10 the worst pain/stiffness imaginable. The anatomical location of pain and stiffness were elicited using two separate body manikins [9]. Participants were also asked whether they had difficulty raising their arms above their head, without reference to whether pain, stiffness or both limited such movement. Patients’ function in undertaking their usual abilities in the past week (e.g. get in and out of bed) was assessed using the modified Heath Assessment Questionnaire (mHAQ) [10].

The baseline questionnaire also collected information on sample characteristics, including age, gender, body mass index (BMI, calculated from self-reported height and weight) and neighbourhood level of deprivation was reported using the indices of multiple deprivation (IMD) [11]. The 7-item Generalised Anxiety Disorder (GAD) questionnaire [12] and Patient Health Questionnaire (PHQ-8) [13] were used to assess the presence of anxiety and depression, respectively.

### Statistical analysis

Descriptive statistics were used to summarise the characteristics of the study sample. The mean age (standard deviation (SD)) and gender were reported. Pain and stiffness were dichotomised at the median (inter-quartile range (IQR)) into low (NRS score 0–7) and high (NRS score 8–10) severity. Using manikin data, participants were categorised by quartiles into the number of painful body sites (0–9, 10–15, 16–22 or 23–44), and patients with bilateral shoulder and/or hip pain also identified. Through the same methods, the number of stiff sites was categorised (0–5, 6–11, 12–19 or 20–44), and participants with bilateral hip and/or shoulder stiffness identified [14]. Patients who responded to being asked whether they were able to lift their arms above their head could answer “yes”, “no” or “do not know”. mHAQ scores were categorised as normal (<0.3), mild (≥0.4 to 1.29), moderate (1.3 to 1.79) or severe (>1.8) functional impairment, based on previously validated criteria [15].

To assess the association between pain, stiffness and mHAQ scores, we used linear regression analysis. The relationship between each pain characteristic and functional status (mHAQ score) was initially examined, followed by each stiffness characteristic and functional status. Associations were reported as the regression co-efficient of the mean mHAQ score with 95% CI. Each association was examined through unadjusted analysis, followed by adjustment for age, gender, deprivation status, smoking status, BMI, anxiety and depression.

IMD was categorised into three groups (the 20% least deprived, mid-deprived and 20% most deprived). BMI was categorised into four groups: (i) <24.9 (healthy weight), (ii) 25.0–29.99 (over weight), (iii) 30.0–34.9 (obese) or (iv) ≥35.0 (severely obese). Smoking status was categorised by those who had never smoked, were ex-smokers or current smokers. Anxiety was categorised as none (GAD7 score 0–4), mild (5–9), moderate (10–14) or severe (15–21). Depression was categorised as none (PHQ8 score 0–4), mild (5–9), moderate (10–14), moderately severe (15–19) or severe (20–24) [12, 13].

## Results

### Sample characteristics

A total of 739 patients with PMR were invited into the study, with 652 responding to the baseline survey (adjusted response rate 88.5%). The mean age of responders was 72.6 years (SD 9.0), the majority were female (62.0%), and a quarter were obese (25.8%) (Table 1).

**Table 1:** PMR cohort characteristics (*N* = 652)

Characteristic	N (%)
Age (mean (SD))	72.6 (9.0)
Age (year categories)	
≤ 64	128 (17.4)
65–69	125 (16.7)
70–74	148 (20.1)
75–80	162 (22.0)
80≥	173 (23.5)
Gender (% female)	457 (62.0)
Deprivation status	
Least deprived (20%)	125 (19.8)
Middle (60%)	389 (61.5)
Most deprived (20%)	119 (18.7)
Body mass index	
<25 kg/m^2^	210 (33.8)
25.0–29.9 kg/m^2^	251 (40.4)
30.0–34.9 kg/m^2^	99 (16.0)
≥35 kg/m^2^	61 (9.8)
mHAQ (mean (SD))	0.57 (0.57)
mHAQ (severity categories)	
Normal (<0.3)	269 (42.8)
Mild impairment (≥0.3 to <1.3)	285 (45.4)
Moderate impairment (≥1.3 to <1.8)	50 (8)
Severe impairment (≥1.8 to 3.0)	24 (3.8)
Anxiety (GAD-7) (n, %)	
None (0–4)	397 (65.2)
Mild (5–9)	132 (21.7)
Moderate (10–14)	49 (8.0)
Severe (15–21)	31 (5.1)
Depression (PHQ-8) (n, %)	
None (0–4)	318 (52.9)
Mild (5–9)	152 (25.3)
Moderate (10–14)	70 (11.7)
Moderately severe (15–19)	38 (6.3)
Severe (20–24)	23 (3.8)

The median NRS severity score for both pain and stiffness was 8 (IQR 7,9). Approximately half of newly diagnosed PMR patients reported pain (47.1%) in ≥16 body sites or stiffness (47.1%) in ≥12 sites. Nearly two-thirds of responders were unable to raise their arms above their head (64.2%).

The mean mHAQ score was 0.57 (SD 0.57), with 269 (42.8%) responders categorised as having normal functioning with no impairment, 285 (45.4%) had a mild functional deficit, 50 (8.0%) had moderate and 24 (3.8%) had a severe functional deficit. The percentage of responders with a score denoting moderate or severe clinically defined anxiety was 13.1%, those with moderate or severe depression was 21.8%.

### Association between pain and function

In the unadjusted model, the mean mHAQ score was 0.24 (95% CI 0.15–0.34) points higher (indicating poorer functioning) in those with a high pain severity (NRS >8) compared to those with a low pain severity. When linear regression analysis was adjusted for age, gender, BMI, deprivation status, smoking status, anxiety and depression, this was attenuated to 0.20 (0.10 to 0.28) and remained significant (Table 2).

**Table 2:** Association between characteristics of pain and stiffness and functional status

		mHAQ Score	
		Regression coefficient (95% confidence interval)	
	N (%)	Unadjusted	Adjusted*
General pain and stiffness			
Pain rating			
Low (0–7)	217 (33.9)	0	0
High (8–10)	423 (66.1)	**0.24 (0.15 to 0.34)**	**0.20 (0.10 to 0.28)**
Number of painful sites			
0–9	180 (27.6)	0	0
10–15	165 (25.3)	0.38 (−0.08 to 0.16)	0.05 (−0.07 to 0.16)
16–22	144 (22.1)	**0.17 (0.04 to 0.29)**	**0.13 (0.01 to 0.25)**
23–44	163 (25.0)	**0.27 (0.15 to 0.39)**	**0.18 (0.06 to 0.29)**
Stiffness rating			
Low (0–7)	255 (39.7)	0	0
High (8–10)	387 (60.3)	**0.23 (0.14 to 0.32)**	**0.18 (0.09 to 0.26)**
Number of stiff sites			
0–5	181 (27.8)	0	0
6–11	164 (25.1)	**0.18 (0.06 to 0.30)**	**0.14 (0.03 to 0.26)**
12–19	157 (24.1)	**0.22 (0.09 to 0.34)**	**0.17 (0.05 to 0.29)**
20–44	150 (23.0)	**0.31 (0.19 to 0.43)**	**0.19 (0.08 to 0.31)**
Localised pain and stiffness			
Can you raise your arms above your head?			
Yes	184 (28.6)	0	0
No	413 (64.2)	**0.28 (0.18 to 0.38)**	**0.19 (0.10 to 0.28)**
Do not know	46 (7.2)	0.17 (−0.02 to 0.35)	0.06 (−0.12 to 0.23)
Bilateral shoulder pain			
No	83 (12.7)	0	0
Yes	569 (87.3)	**0.22 (0.09 to 0.36)**	**0.18 (0.05 to 0.31)**
Bilateral hip pain			
No	237 (36.3)	0	0
Yes	415 (63.7)	0.71 (−0.22 to 0.16)	0.80 (−0.01 to 0.17)
Bilateral shoulder stiffness			
No	184 (28.2)	0	0
Yes	468 (71.8)	**0.19 (0.09 to 0.28)**	**0.10 (0.01 to 0.20)**
Bilateral hip stiffness			
No	313 (48.0)	0	0
Yes	339 (52.0)	**0.12 (0.03 to 0.20)**	0.07 (−0.02 to 0.15)

**Bold** = significant difference. *Adjusted for age, gender deprivation, BMI, smoking status, anxiety and depression and current prednisolone dose

Unadjusted analysis showed an association between patients with an increased number of pain sites (16–22 pain sites 0.17 (0.04 to 0.29); 23–44 sites (0.27 (0.15 to 0.39)) and poorer functional status compared to those with 0–9 pain sites. When adjusted, this was attenuated to 0.13 (0.01 to 0.25) for 16–22 sites and 0.21 (0.06 to 0.29) for 23–44 sites, though remained significant.

The relationship between bilateral shoulder pain and function also showed a significant association. In the unadjusted model, the mean mHAQ score was 0.22 (0.09 to 0.36) points higher in those with bilateral shoulder pain compared to those without. When linear regression analysis was adjusted, this was attenuated, but remained significant (0.18 (0.05 to 0.31)). There was no statistically significant association between bilateral hip pain and functional status after adjustment.

### Association between stiffness and function

In the unadjusted model, the mean mHAQ score was 0.23 (95% CI 0.14 to 0.32) points higher (poorer functioning) in those with a high stiffness severity (NRS >8) compared to those with a low stiffness severity. When linear regression analysis was adjusted, this remained significant 0.18 (0.09 to 0.26). We also found that as the number of stiffness sites increased, there was an association with poorer function. In the unadjusted analysis, there was an association between 6 and 11 stiff sites (0.18 (0.06 to 0.30)), 12–19 stiff sites (0.22 (0.09 to 0.34)) and 20–44 stiff sites (0.31 (0.19 to 0.43), which remained significant after adjustment (6–11 sites 0.14 (0.03 to 0.26); 12–19 stiffness sites 0.17 (0.05 to 0.29) and 20–44 stiffness sites to 0.19 (0.08 to 0.31) (Table 2).

Though there was an unadjusted association between the presence of either bilateral shoulder stiffness (0.19 (0.09–0.28)) or bilateral hip stiffness (0.12 (0.03–0.20)) and the functional status of patients with PMR, this was only retained for bilateral shoulder stiffness after adjustment.

Finally, an association was examined between the PMR patients’ ability to raise their arms above their head and functional status. Unadjusted analysis showed that those unable to raise their arms above their head were significantly more likely to have poorer mHAQ scores than those who could raise their arms above their head (0.28 (0.18–0.38) and this association was retained after adjustment (0.19 (0.10 to 0.28)).

## Discussion

Our research used baseline data from an inception cohort of primary care patients with newly diagnosed PMR to better understand the relationship between pain, stiffness and the patients’ ability to function in their daily activities. Our results demonstrate that in the earliest stages of PMR, over half of patients have some degree of functional limitation. Those PMR patients with high pain or stiffness severity, a high number of painful or stiff body sites and those with limitations in the shoulders report significantly poorer functional status.

The ability of newly diagnosed PMR patients to conduct their daily activities has not previously been examined within a primary care population. However, two prospective cohort studies recorded baseline mHAQ scores in secondary care populations of patients with new-onset PMR. Matteson et al. reported a median (IQR) mHAQ score of 1.1 (0.8, 1.7) in a US sample (*n* = 85) [16] and Dasgupta et al. reported a median mHAQ score of 1.1 in a sample from the UK (*n* = 125) [17]. Our sample of primary care PMR patients reported a lower mean score (better function) than these small samples from secondary care. Though this variation may potentially be a result of study design and sample differences, we would expect those patients’ with PMR in the secondary care system to be those with more severe, atypical or long-standing problems and therefore potentially poorer ability to function in daily living [7]. However, while these overall scores do differ, when categorised by severity of functional difficulties, this previous research and our own all found impairment in function for the majority of newly diagnosed PMR patients to be “mild” (mHAQ score < 1.3).

Our sample of patients with PMR also has improved functional capacity compared to patients with other rheumatic conditions. Pincus et al. (1999) reported mean mHAQ scores for patients with RA, fibromyalgia, osteoarthritis or vasculitis from secondary care to be 1.73, 1.64, 1.52 and 1.39, respectively. These scores suggest that patients with prevalent rheumatological conditions are experiencing a “moderate” impact on function, compared to the “mild” impact seen within our sample, even though these samples were generally 20 years younger than our participants [18]. Therefore, it appears that patients with newly diagnosed PMR from primary care are either not experiencing functional problems, or if so, these typically represent a mild impairment. Those from secondary care have, on average, a higher mHAQ score, but function appears less impaired than for those with other rheumatic conditions. Therefore, those patients with newly diagnosed PMR reporting severe or widespread pain or stiffness in primary care may represent a subset of patients who might warrant additional follow-up and support to ensure improvement or perhaps interventions (i.e. referral to physiotherapy or occupational therapy) to improve function if function remains impaired. However, our study demonstrates the wide spectrum of PMR severity which GPs see. The more severe cases are not solely seen in secondary care and this poses some difficulty for GPs in selecting those in need of additional support. This also indicates the collaborative role which should (and can) happen between GPs and rheumatologists. Such identification is not only important for the patient; loss of function poses a significant cost, both socially and economically. It forces increased formal and informal health care costs, it increases the likelihood of additional injuries and increased consultations to GP practices [19]. Future research may consider how best to use the questions GPs already likely ask PMR patients during consultations to determine which patients are likely to require help with regard to function. For example, asking whether a patient can raise their arms above their head may be a useful, quick and simple marker of overall functional limitations.

### Strengths & limitations

This study is the first inception cohort study in primary care to examine newly diagnosed PMR patients. Response rates were excellent and as this data is based on incident PMR patients, recall bias is minimised. We were also able to recruit a large sample of patients across a wide geographical area and therefore our results are generalisable to PMR patients from across England who consult in primary care.

Our limitations include the use of routinely collected data, including the diagnosis of PMR. It is possible that some people referred into the study may not have been considered to have PMR given time or if reviewed by a rheumatologist and therefore includes the possibility that some of our PMR patients were later re-diagnosed with a different condition. However, our provision of information to GPs through the recruitment template, the balance of our samples’ demographics, in addition to large proportions with bilateral shoulder and/or hip pain and stiffness, adds further confidence of the accuracy of the PMR diagnosis, rather than alternative conditions where patients’ experience widespread pain or stiffness. Furthermore, this sample represents the cross-section of individuals that GPs diagnose and manage with PMR in UK primary care.

### Conclusion

Our study demonstrates that the majority of patients with polymyalgia rheumatica experience no, or mild functional limitations when newly diagnosed in primary care. However, a significant association does exist between widespread, severe pain and stiffness and functional status. As the ability to function in daily activities is so important to patients, primary care is well placed to examine this subset further. At the point of diagnosis, early specific assessment of function (for example, a simple question on ability to raise their arms already being asked by the GP) may be needed to help to prioritise and assess patients further for more intensive support and treatment.
